# Functional Land Management: Bridging the Think-Do-Gap using a multi-stakeholder science policy interface

**DOI:** 10.1007/s13280-017-0983-x

**Published:** 2017-11-24

**Authors:** Lilian O’Sullivan, David Wall, Rachel Creamer, Francesca Bampa, Rogier P. O. Schulte

**Affiliations:** 1 Teagasc, Crops, Environment and Land Use Programme, Wexford, Johnstown Castle Y35 Y521 Ireland; 2 Farming Systems Ecology Group, Wageningen University and Research Centre (WUR), 6708 PB Wageningen, The Netherlands; 3 Soil Biology and Biological Soil Quality Group, Wageningen University and Research Centre (WUR), PO Box 47, Droevendaalsesteeg 4, 6708 PB Wageningen, The Netherlands; 4 Faculty of Economics and Social Development, Latvia University of Agriculture, Jelgava, Latvia

**Keywords:** Functional Land Management, Policy framework, Soil functions, Stakeholder workshops, Sustainability, Think-Do-Gap

## Abstract

Functional Land Management (FLM) is proposed as an integrator for sustainability policies and assesses the functional capacity of the soil and land to deliver primary productivity, water purification and regulation, carbon cycling and storage, habitat for biodiversity and recycling of nutrients. This paper presents the catchment challenge as a method to bridge the gap between science, stakeholders and policy for the effective management of soils to deliver these functions. Two challenges were completed by a wide range of stakeholders focused around a physical catchment model—(1) to design an optimised catchment based on soil function targets, (2) identify gaps to implementation of the proposed design. In challenge 1, a high level of consensus between different stakeholders emerged on soil and management measures to be implemented to achieve soil function targets. Key gaps including knowledge, a mix of market and voluntary incentives and mandatory measures were identified in challenge 2.

## Introduction

The growing demands on land and soil globally add ever growing complexity to policies aimed at agricultural and environmental land management. Agriculture is faced with the challenge of increasing primary productivity to meet the rising global demand for food security (Alexandratos and Bruinsma 2012). With United Nation (UN) population estimates of between 9.4 and 10 billion for 2050, increasing to between 10 and 12.5 billion by 2100 (UN 2015a), food security continues to be a priority on the political agenda. At the same time, society expects that any emphasis on increasing agricultural output is met with an equal emphasis on sustainability (Garnett et al. 2013). The intensification of agriculture, while not always, has often been associated with negative environmental consequences. Agriculture is the main source of nitrate and phosphate pollution to water (OECD 2001; FAO 2003) and is a major source of methane and nitrous oxide to the atmosphere with Agriculture, Forestry and Other Land Use (AFOLU) responsible for just under one-quarter of anthropogenic greenhouse gas emissions (FAO 2003; Smith et al. 2014). As well as contributing to climate change, agriculture is affected by it. While warmer temperatures can support the growth of specific crops in certain part of the world up to a point, if temperatures exceed an optimal level or if there are insufficient water and nutrients, a decrease in yields is anticipated, associated with climate change (FAO 2016). In relation to soil, the majority of the world’s soil resources are in fair, poor or very poor condition, while one-third of land is moderately to highly degraded (FAO and ITPS 2015).

A response to these challenges is reflected in the Sustainable Development Goals (SDGs). Building on the forerunning Millennium Development Goals, the SDGs outline the action plan to be implemented by all countries and include four targets specifically citing soil (2.4, 3.9, 12.4 and 15.3) with two other targets that focus on land and soil functions. By 2030, these targets seek to progressively improve soil quality, reduce soil pollution and contamination and to restore degraded soils (UN 2015b). A global literature review of the relationship between soils and ecosystem services is presented by Adhikari and Hartemink (2016), and despite some emphasis on the role of soils in the contribution to ecosystem services (Blum 2005; EC 2006; Haygarth and Ritz 2009; Bouma 2014; Bouma et al. 2015), overall, soil is generally an overlooked component in studies related to ecosystem services and policy decision making (Hewitt et al. 2015). Within the European Union (EU), the withdrawal of the proposed Soil Framework Directive in 2014 highlighted the need for stakeholders and lobby groups to think differently about soils (Bouma and Montanarella 2016). The proposed Soil Framework Directive emphasised the need for soil protection which led to resistance from key agricultural stakeholders and arguably distracted from efforts to include soil functions in the development of land use and management policies (Robinson et al. 2012; Adhikari and Hartemink 2016).

## Functional Land Management: the concept

In response, Schulte et al. (2014) proposed the Functional Land Management (FLM) framework. This utilitarian framework seeks to optimise the supply of soil functions from the land through sustainable use of Europe’s soil resource. The core concept of FLM is the multi-functionality of soils, which is that all soils deliver multiple functions simultaneously, but that some soils are better at the delivery of certain soil-based ecosystem services over others. The subset of ecosystem services that rely on soil and land use for their delivery are recognised as “soil functions” (Bouma 2014) and were first described in the European Commission Thematic Strategy for Soil Protection (EC 2006). FLM focuses on the five soil functions that are delivered through agricultural landscapes: (1) primary productivity, (2) water purification and regulation, (3) carbon cycling and storage, (4) habitat for biodiversity and (5) recycling of (external) nutrients/agro-chemicals. The EU LAND Management: Assessment, Research, Knowledge base (LANDMARK) project (SFS-04-2014-soil quality and function) is quantifying the supply of soil functions across Europe. This quantification will recognise the variable intrinsic capacity of the soil under different land uses and management practices to simultaneously deliver soil functions to a greater or lesser extent (Coyle et al. 2016). It will therefore be determined by soil properties, environment, land use and soil management practices.

While the supply of soil functions depends upon biophysical criteria, environment and management, within the FLM framework, the contrasting demands for soil functions are framed as EU policies. For example, demands for the water purification and regulation function include the EU Water Framework Directive that requires all water bodies to be of ‘good’ ecological status (EU 2000) and the Nitrates Directive that indicates that groundwater nitrates-N (NO_3_-N) concentrations must not exceed 11.3 mg per litre (EU 1991). Altogether, FLM has the potential to combine inter- and trans-disciplinary research, along with a more holistic approach to the land base representing an integrator for sustainability policy. Integrated issues are complex, both to understand and to manage, and are associated with uncertainties that must be characterised in advance, so that potentially irreversible or long-term negative consequences can be avoided, but this relies on an increased knowledge demand (EC 2012a).

## Functional Land Management: from research to implementation

Several research studies have thus far demonstrated the potential of the FLM framework. Coyle et al. (2016) extended the FLM framework to show the multi-functional capacity of soils for the European Atlantic pedo-climatic zone. A matrix was developed based upon land use and soil types clustered by drainage class to show the consequential changes to the capacity of the five soil functions including the potential trade-offs for individual functions and the overall impact on the multi-functional capacity (suite of five functions) of soil. To demonstrate this, O’Sullivan et al. (2015) provided a first example of the application of the FLM for policy decision making. The trade-offs between the soil functions ‘primary productivity’ and ‘carbon cycling and storage’ in response to the intervention of land drainage systems applied to ‘imperfectly’ and ‘poorly’ draining managed grasslands were explored. These trade-offs were expressed as a function of the nominal price of ‘Certified Emission Reductions’ and were characterised spatially using ArcGIS to account for spatial variability of the supply of soil functions. The results highlighted large geographic variation in the environmental cost:agronomic benefit ratio. This example demonstrated the potential of FLM to facilitate a shift away from blanket policies to develop policies that can be tailored to contrasting biophysical environments that can be more effective at the prioritisation of contrasting soil functions. To explore the FLM framework further, Valujeva et al. (2016) used a non-spatial land use model to assess the supply of soil functions for contrasting soil drainage and land use categories under different optimisation scenarios. As additional soil functions were added, the management requirements became more complex. This research highlighted a challenge for policy makers: in order to meet current and future agronomic and environmental targets, the supply of each soil function needs to be managed at the spatial scale at which the corresponding demand manifests itself, which may range from farm to national scale. As well as the spatial mismatch that exists between supply and demand, Valujeva et al. (2016) also emphasised a need to consider the temporal mismatch between the supply and demand for soil functions. These modelling studies are now underpinned by the Soil QUality Assessment and REsearch (SQUARE) project (DAFM Project Reference No: 13S468), which encompasses a national level field campaign that will provide a baseline of the delivery of the five soil functions for grassland management systems in Ireland.

## Aims and objectives

Part of the challenge in developing the FLM concept rests in addressing how this research framework can be translated in practice and be implemented in reality. Based on FLM, governance instruments for managing the soil and land resource sustainably must account for the differences between soils and landscapes. Schulte et al. (2015) identified 15 existing governance instruments (divided into market, mandatory and voluntary) to manage soil functions from local to national/EU scale. They concluded that further research should explore if these could be realigned so that the differences between soils and landscapes are included (Schulte et al. 2015). Importantly, this does not necessarily equate to a legislative zoning of land management practice, but seeks to promote incentives that foster action to optimise the functionality of our land based on the soil resource. Given the long history of incentivisation within the EU, Schulte et al. (2015) conclude that in principle mechanisms for incentivisation are already in place and could be adapted for the implementation of FLM. The challenge then is how best to realign instruments to translate the research into practice. Currently, a gap exists between the scientific design of optimised land management as conceptualised in FLM and the implementation in practice. This research aims to bridge this knowledge gap—here called the Think-Do-Gap. Specifically, the aims and objectives of this work are to:

1. Design an optimised catchment management plan to hypothetically reflect the implementation of FLM at catchment scale.
2. Identify the gaps to implementation of catchment design/FLM.

## The Functional Land Management catchment challenge: methods

We developed the ‘catchment challenge’ workshop method in order to bridge the Think-Do-Gap on sustainable land management, i.e. the discrepancy between the scientific design of FLM and the implementation in practice. The catchment challenge is designed as a multi-stakeholder science policy interface to support the translation of research to governance with the overall aim of landscape implementation of the FLM concept. The catchment challenge method can be used to harvest information and data on the gaps, actors and instruments necessary to implement FLM. The challenges are intended to get stakeholders to design a catchment with consideration of the need to supply all soil functions within a landscape. Stakeholders must match the supply of soil functions with the societal demand for soil functions through use of land use, land use change and land management options. With the exception of workshop No. 7, where farmers completed an outdoor workshop assessing three soil profiles, the workshops (approximately *n* = 235 participants) included the same core focus of designing the management and implementation of an optimised landscape (Table 1).

**Table 1:** Stakeholder workshops including institutional representation of eight stakeholder workshops, approximate number of participants and the format, facilitated in Ireland between 2014 and 2016

ID. Workshop	Stakeholders and institutional representation	No. (approx.)	Format		
1. Irish Soil Information System Launch 2014	Irish Department of Agriculture, Irish Environmental Protection Agency, Teagasc Agriculture and Food Development Authority, European Commission JRC, Universities, Students, Farming Press	19	a	Entry ranking of soil functions	Catchment challenges: (1) unconstrained design and (2) pathways
2. Crops and Nutrition Course 2014	Teagasc Advisory and Research, Private Advisory, Industry agrochemical/fertiliser, Farmers, Students	26	a	Entry ranking of soil functions	Catchment challenges: (1) unconstrained design and (2) pathways
3. LANDMARK Horizon 2020 Project Launch 2014	Teagasc; Universities: Denmark, Hungary, United Kingdom, Belgium, Romania, Sweden, Italy; European Commission JRC Italy; RIVM Netherlands; Chambers of Agriculture France; Chamber of Agriculture of Lower Saxony Germany; AGES Austria; INRA France; Institute of Social Science Chinese Academy of Sciences China; ETH Zurich Switzerland, Jozef Stefan Institute Slovenia	30	b	–	Catchment challenges: (1) Unconstrained design and (2) based on LANDMARK Pillar II (monitoring)
4. Catchment Science week 2015	Irish Department of Agriculture, Irish Environmental Protection Agency, Teagasc Agriculture and Food; AFBI Northern Ireland; European Commission JRC; Universities/Students (United Kingdom, New Zealand), Farmer, Consultancy, County Council	28	c	–	Catchment challenges: (1) unconstrained design and (2) pathways
5. Agricultural Catchments Programme 2015	Teagasc Advisory, Research, Student Researchers, Farmer, Farm Management	16	d	Entry and exit ranking of soil functions	Catchment challenges: (1) unconstrained design and (2) pathways
6. Co-operative Industry 2015	Processor Executive, Farmer Co-op Board Member, Processor Sustainability, Processor, Processor Nutrition, Processor Quality Control, Farm Sustainability Manager, Veterinary	22	d	Entry and exit ranking of soil functions	Catchment challenges: (1) unconstrained design and (2) pathways
7. Farming Group 2015	Farmers—tillage	30	e	Survey instrument	Profile pit assessments
8. International Farmer Scholarship 2016	International Researchers, Farmers, Students with Guest Panellists including NGO, Farmer, Department of Agriculture Food and the Marine, Northern Ireland EPA, Academic Policy Analyst, Co-operative Sustainability Manager	80 +	f	Role play	Catchment challenges: (1) unconstrained design and (2) pathways

Workshop participants represented a broad diversity of stakeholders including the academic and research community (national and international), farming community, public sector, private sector, processors including co-operatives, policy makers, advisory and lobby groups including non-governmental organisations (Table 1). Collectively, these stakeholders represent a broad cross-section of society with the potential to influence the implementation of FLM at multiple scales.

**Fig. 1:**
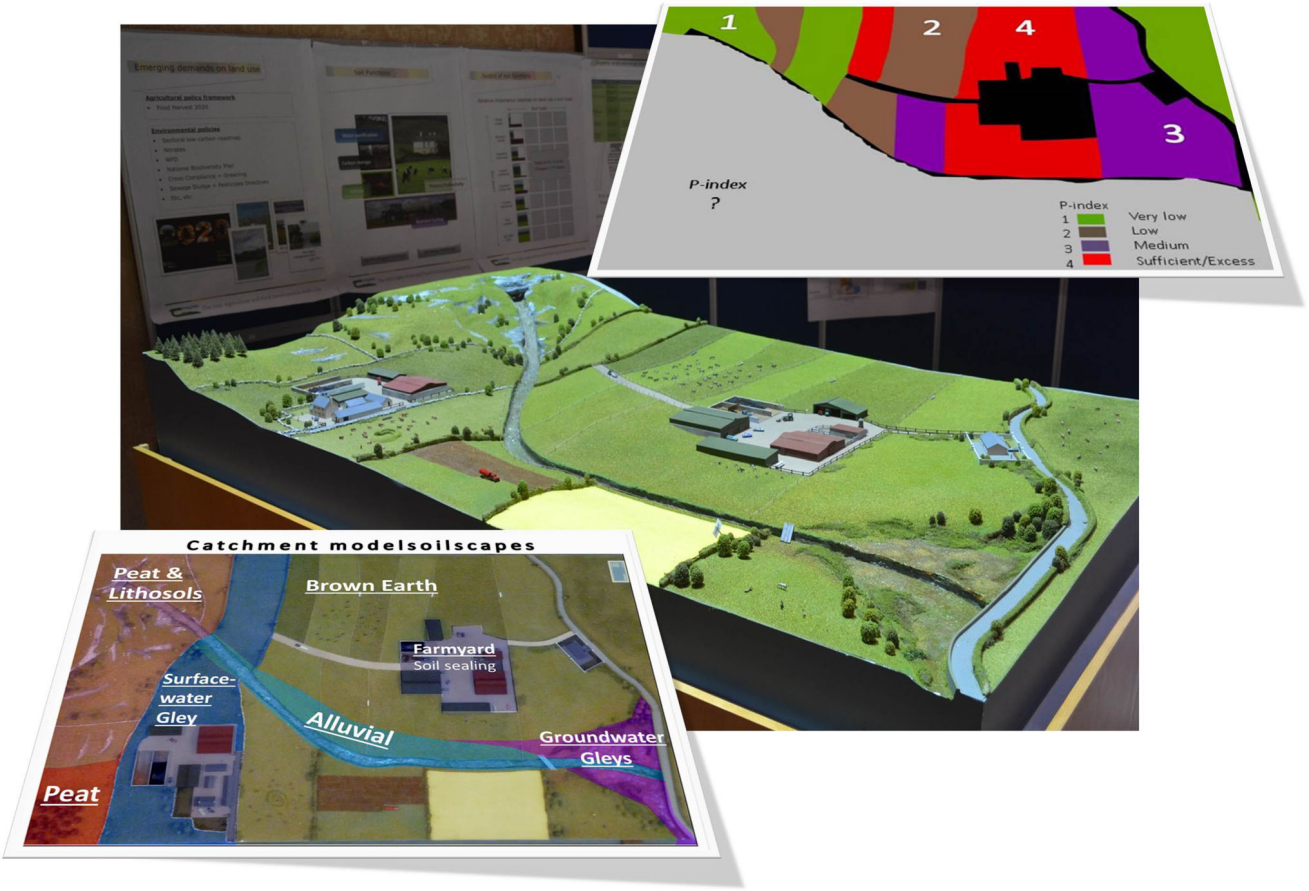
Catchment model used for FLM interactive learning and knowledge co-production (centre); the landscape model is described in terms of soil types (bottom left), and soil test phosphorus (P) status (top right)

At the outset of the workshops, the key concept of FLM and the multi-functional capacity of soil as defined by Schulte et al. (2014) were explained with a poster series and a catchment model (Fig. 1) which provided the centrepiece of the workshop discussion. The challenges were as follows:

1. To increase the supply of two of the soil functions in the catchment to meet demands: (a) the primary productivity function by increasing milk production by 50% on one of the catchment farms (Ann’s farm—described below). This challenge is consistent with the demand target outlined in Ireland’s Food Harvest 2020 policy document (DAFF 2010); (b) the water quality function, by improving the water quality status from Q3 (moderate) to Q4 (good) under the EU Water Framework Directive (EU 2000). In addition, the delivery of the other soil functions must not to be reduced within the catchment design.
2. To identify gaps, pathways and policies to facilitate the catchment designed in challenge 1 to become a reality. Information on existing mechanisms was presented along with the relative scale and respective function(s) that they apply to see Schulte et al. (2015).

Challenges were conducted in smaller breakout groups and A0 maps along with some options for land use and management on small pieces of paper were provided for the breakout groups. This facilitated groups to visually display the options decided/discussed being presented by their rapporteur for challenge 1. In challenge 2, the same breakout groups had to identify the gaps, pathways and policies to meet societal expectations of the land base which were also reported back to the group.

The farmers presented in this fictitious scenario represent two very different realities/systems with polarised ambitions for their farming futures. Ann is a young progressive, educated farmer, who is anxious to intensify her dairy business, whereas John is a middle-aged farmer, with off-farm income, who operates a suckler beef enterprise and is seeking to reduce the time commitment of his farm operation. Their commonalities include that both share a boundary with the river as well as grazing rights on the catchment hill. John has not had his soils sampled for nutrient analysis, whilst Ann has full knowledge of her soil resource. Soils found in this catchment range from very wet Peats (Histosols) and shallow soils (Histic Lithosols) to deep Surface-water Gleys (Stagnosols) and free-draining Brown Earths (Haplic Cambisols) found in the catchment heartland. The Alluvial (Fluvisols) soils bordering the river are frequently waterlogged and are associated with poor drainage and poor trafficability with Groundwater Gleys (Gleysols) found on the lowest ground due to a high water table (Fig. 1, bottom left). In relation to the phosphorus (P) status of the soils in the catchment, a lack of soil analysis on one-half of the catchment means that the P-index value is unknown (Fig. 1, top right). Elsewhere, the upland areas reflect very low P-status in comparison to the fields around Ann’s farmyard, where a soil test P-index of four is indicative of a potential excess of P.

A photographic image of the catchment model was taken and using ArcGIS software the landscape was divided into polygons representative of different soil types typical of a catchment catena. These polygons were coloured in different colours after which the image was exported and saved in Microsoft PowerPoint 2010. For demonstration purposes, polygons were vertically projected by soil types, superimposed onto the physical catchment, whilst the workshop moderator described that particular soil type/part of the landscape. The PowerPoint soil map was printed out in A0 for workshop groups. The P-Index printouts were similarly created and printed in A4. As these workshops took place in Ireland, the catchment and fictitious scenario presented are typical for an Irish context but can be customised regionally based on location according to climate, pedology, land use and management as has been done in partner countries in the LANDMARK project.

Results from challenge one were recorded on A0 sheets with citations shown at workshop level. The results from challenge 2 were recorded on flip charts with additional note taking during open discussion.

Participants were asked to complete a ranking exercise where stakeholders indicated their prioritisation of soil functions. In workshops one and two, this was an ordinal ranking from one to five, representing the least to the most important respectively, for the five soil functions. In workshops 5 and 6, the ranking exercise was adapted so that stakeholders could allocate 15 points across the five functions, with a maximum of five for any one function. This allowed instances where a soil function has an equal weighting with another function, or where a soil function is not a priority at all, to be identified. Also at workshops 5 and 6, the same ranking exercise was completed at the end of the workshop, to capture any changes in prioritisation. Data were averaged by stakeholder group with means shown in radar diagrams. A *t* test to assess differences in before and after ranking by soil function was completed using Statistica with a significance value of *p* < 0.05.

Results recorded from the survey instrument in workshop 7 were cleaned, coded and input into a Microsoft access database.

## Results and discussion

### Challenge 1

The results from catchment challenge 1 are shown in Table 2 with breakout group responses presented at workshop level clustered into three categories: land use change, land management practices and knowledge intensification measures. Across all workshops, the top five options proposed were afforestation, the use of buffer strip/riparian zones, soil sampling and analysis, targeted inorganic nutrients and targeted slurry/organic amendments.

**Table 2:** Options proposed for an optimised catchment design to achieve an increase in primary productivity (50% on one farm) and an improvement in the water quality function (from Q3 to Q4 under the Water Framework Directive) while maintaining the carbon storage and cycling, habitat for biodiversity and nutrient recycling functions. Results are clustered into land use change options, land management practices and knowledge intensification measures. Options cited are highlighted in grey, whereas a white box indicates that the option was not proposed

In relation to land use changes, afforestation, bioenergy crops, conversion to grassland and conversion to dairy were the options most frequently proposed in the optimised designs. Land management practices related to grass management were repeatedly cited, of which extending the grazing season was considered most important towards meeting the primary productivity target (*n* = 6). Buffer strips or fencing off high-risk areas were unanimous solutions (*n* = 7) towards the protection and improvement of water quality, targeted in areas considered to be critical source areas. The options of soil analysis, targeted organic nutrient amendments and inorganic nutrient management plans to augment nutrient efficiency and environmental gains were important having been cited by all groups. Other efficiency gains proposed, related to the specialisation of operations, such as the contract rearing of heifers, or the leasing of commonage shares to allow one farmer to take sole responsibility for sheep rearing on the hill areas of the catchment. Importantly, many of the options proposed assume a level of education and it is therefore an important consideration that if farmers are to deliver optimised management for soil functions, there is an implicit knowledge demand.

Despite the diversity of stakeholders, there was a high level of agreement in relation to the development of a catchment management plan to achieve the optimal delivery of soil functions and to meet the targets of challenge 1. Stakeholders were able to collectively achieve consensus about how to design an optimised catchment in an unconstrained scenario. All groups used the information in relation to soil types to design their ideal catchment. This signals another important consideration related to implementation of FLM: knowledge gaps related to soil and land use could impact local level decision making and could result in suboptimal decision making. Soil analysis and better nutrient management plans were proposed by all groups who completed workshop challenge 1 (*n* = 7), reflecting the importance of these options. The knowledge gap associated with farmer John’s lack of soil analysis represented a barrier to the implementation of optimised catchment management. This lack of knowledge was further found to be associated with reduced economic opportunities for farmer-to-farmer collaboration, with a nutrient trading scheme cited as one potential missed opportunity in the scenario. A shared finding for all groups was that implementation of the optimised catchment extends beyond the farm and that farmer collaboration is a key requirement for achieving optimised landscape management. This is endorsed by the fact that several of the proposed measures rely on farmer-to-farmer or farmer-to-business interactions, such as contract heifer rearing, leasing land or nutrient trading as some cited examples.

### Challenge 2

**Fig. 2:**
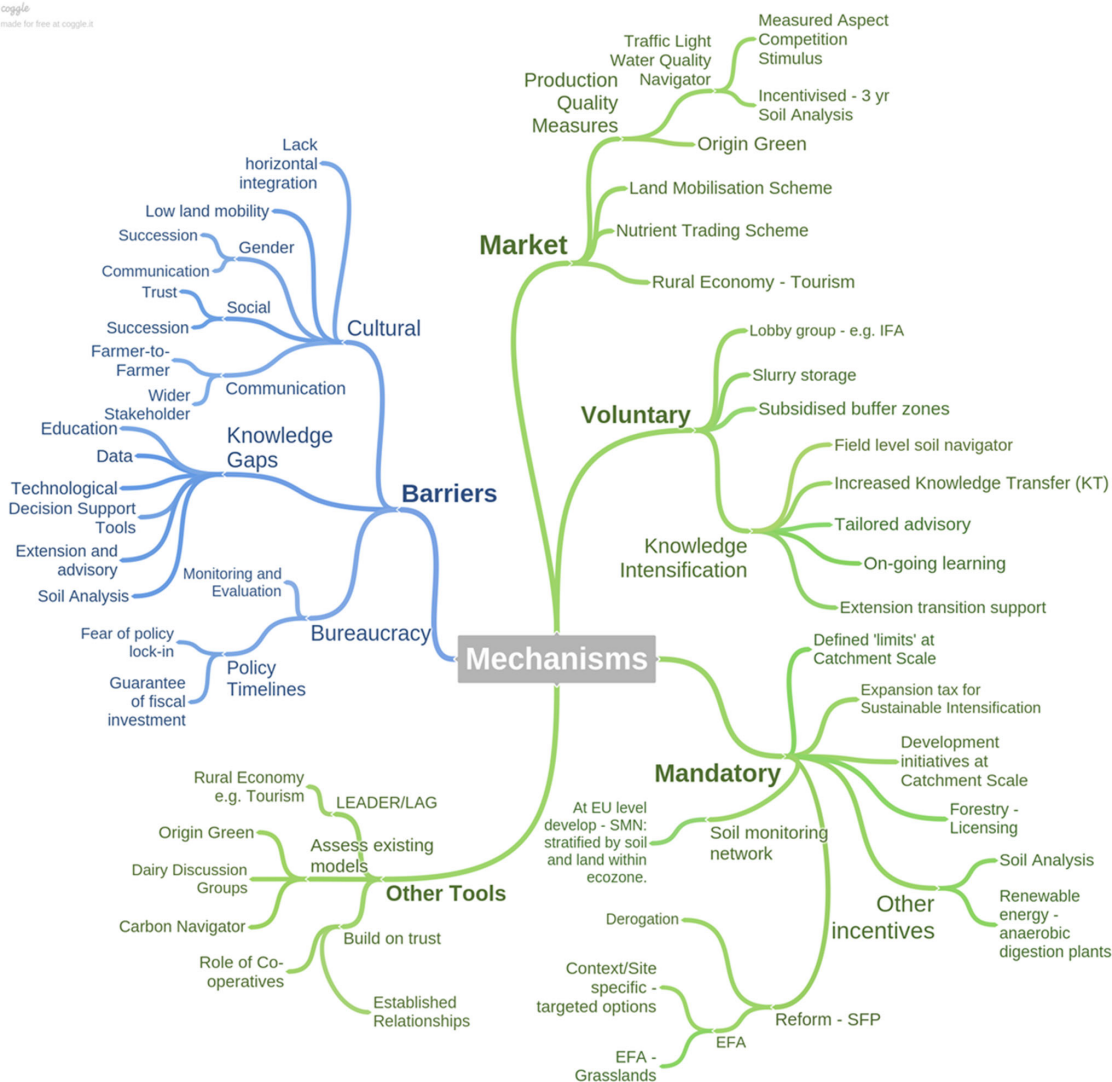
Gaps that inhibit optimised land and soil management, with barriers shown in blue and the policy instruments required to steer change shown in green (using http://coggle.ie/)

For challenge 2, participants were asked to identify the governance tools that might be necessary to achieve the catchment design proposed in challenge 1. These could include policy tools or market instruments, and participants were advised that they could utilise existing tools or develop new tools where a gap was found to exist. The key gaps and mechanisms for the achievement of the catchment management design from challenge 1 identified are shown in Fig. 2.

**Fig. 3:**
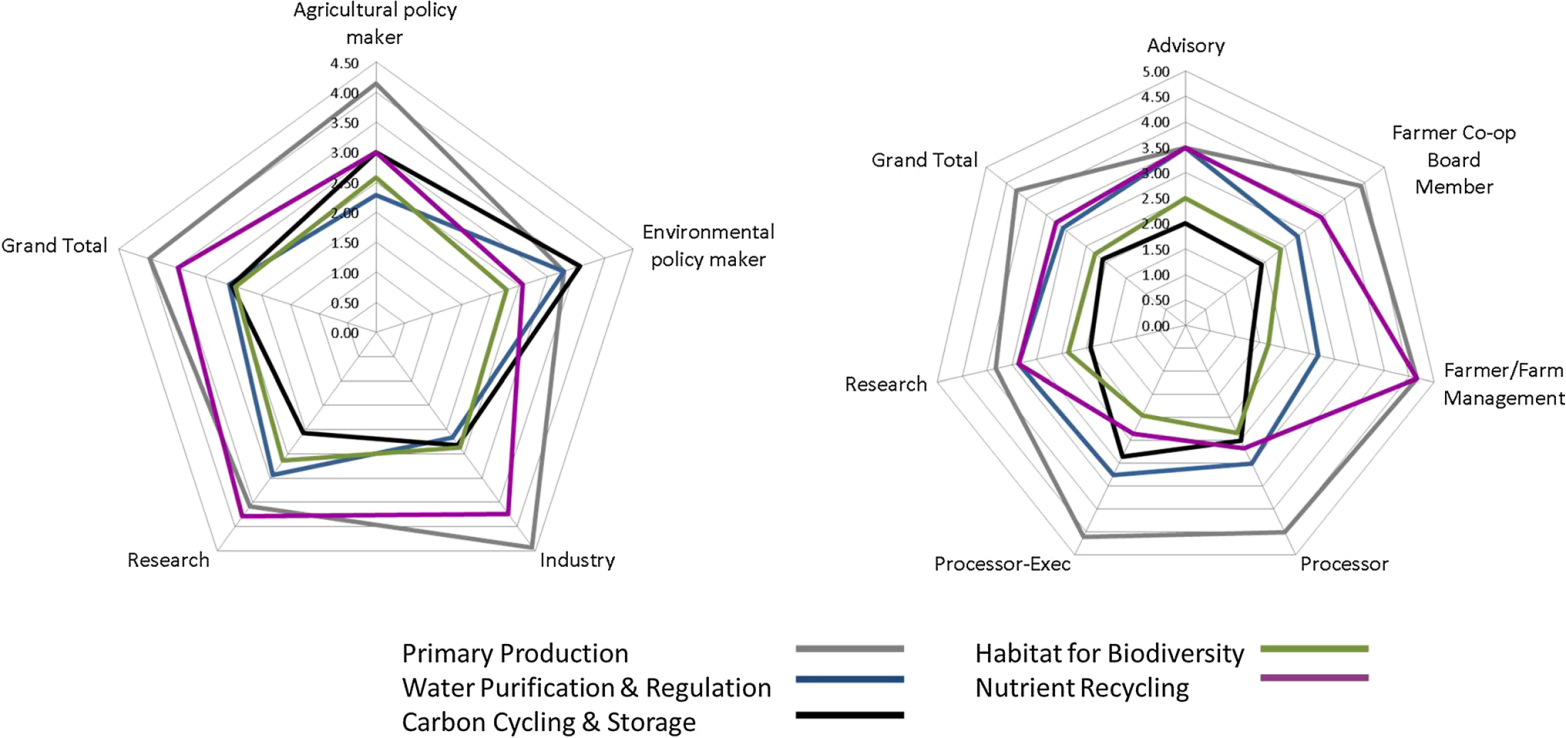
Prioritisation of soil functions by stakeholder groups. Radar diagram on the left representing the results from workshops 1 and 2, radar on the right from workshops 5 and 6. The first graph shows the ranking based from one to five for the soil functions. The second graph represents an optimisation of the ranking exercise where stakeholders are asked to rank five soil functions with a maximum of five for any one functions thereby highlighting instances where functions may not represent a priority at all. The second method has been adopted for use within LANDMARK workshops

In relation to gaps, cultural barriers were considered important with gender, social and communication gaps all hindering within-catchment level cooperation. Discussions around bureaucratic issues, including policy timelines, highlighted a clear misalignment, whereby farmers’ fear of “policy lock-in” was in sharp contrast to policy makers and their preference for longer-term measures to guarantee the fiscal investment of policy incentives. This finding potentially indicates that the threshold for the uptake of voluntary policies could be raised. From a policy perspective, this indicates that higher fiscal incentives could be required for local level implementation. Knowledge gaps emerged as important, cited at all workshops (*n* = 7). Seven out of seven workshops cited that knowledge transfer, farm advisory services and farmer discussions, soil analysis and decision support tools were necessary at farm level. Specifically, more advisory support, training and education were emphasised by all groups. At policy scale, information gaps on the synergies between national level target setting and on-farm management practices were highlighted and are expressive of an on-going requirement to develop pathways that connect the two, cited in five of the workshops—1, 3, 4, 6 and 8.

Concerning mechanisms to overcome gaps to achieve the ideal catchment, suites of market, mandatory and voluntary measures were proposed. Market measures, largely driven by quality production measures were proposed. In Ireland, the green credentials of Irish produce as captured in the “Origin Green” initiative by Bord Bia (Irish Food Board) were highlighted in workshops 1 and 6, as one example whereby synergies could be achieved for producers and policy makers across ministries including agriculture and environment. In workshop 4, another example proposed was the development of a traffic light water quality navigator that could offer value in relation to sustainable branding. Mandatory measures included an expansion tax for sustainable intensification or the inclusion of defined catchment scale limits for environmental indicators. The implementation of Ecological Focus Areas (EFAs) for grasslands was proposed (workshops 2, 4, 5 and 6). A Soil Monitoring Network to afford soil the necessary protection to maintain its sustainability into the future was proposed at national and EU level (workshops 1, 3, 4 and 5). Consequently, options for a soil monitoring network for Ireland have been proposed (O’Sullivan et al., 2017). Related to this, monitoring and evaluation requirements were cited as essential considerations for the deployment of governance tools. Voluntary measures focussed on opportunities for knowledge intensification that included the introduction of a field level “soil navigator” a decision support tool for sustainable soil management, and increased knowledge transfer. The “other tools” cited, mostly referred to existing models, for example the “dairy discussion model” designed for farmer discussion groups. This model is one rural development measure under the ‘Knowledge Transfer and Information Actions’ co-funded by the EU’s European Agricultural Fund for Rural Development (EAFRD), Pillar II of the Common Agricultural Policy and the Irish national exchequer (DAFM 2016). Farmer discussion groups are facilitated by advisors, and information and best practices are shared between farmers. These groups show to have a positive impact on technology adoption and profit levels (Hennessy and Heanue 2012). The dairy discussion model was proposed as having capability to be moulded for multiple farming systems for implementation at catchment scale. In this regard, the role of the co-operatives or the use of established trusted relationships was considered important for supporting farm level change including off-farm interaction.

Targeted policies are designed to pursue particular outcomes applied to identified groups or areas that are most likely to produce the desired outcome (Moreddu 2007). Options to increase targeted policies were discussed, but opinions as to how this could be achieved diverged. “Hard” policy instruments include legally binding rules such as regulations, directives and decisions (EC 2012b). A mapping approach based upon soil types was one such option proposed. A need for more tailored regulation that takes account of soil type and hydrology with respect to N and P losses has previously been identified for Ireland, versus blanket ‘one size fits all’ policies (Buckley 2012). Scientific evidence to support a shift away from blanket policies is essential, as widespread transgressions can emerge where regulation is perceived as unnecessary, resulting in high monitoring and enforcement costs (May and Winter 2001). The FLM approach seeks to respond to this challenge through the integration of policy instruments for multiple soil functions whilst promoting policy design that considers the variation in soil capacity. “Soft” policy instruments are more flexible approaches including recommendations (EC 2012b) and options related to education, knowledge transfer, one-to-one farm visits and discussion groups were proposed.

### Soil functions: prioritisation, ranking and farmer perceptions

Across these stakeholders (workshops 1, 2, 3, 4), on average the primary productivity and the nutrient recycling functions emerged as the highest priorities. An exception to this was the environmental policy makers who prioritised the carbon cycling and storage function ahead of primary productivity which ranked second along with the water purification and regulation functions (Fig. 3 left). Industry stakeholder groups similarly prioritised primary productivity and nutrient recycling, with the exception of the executive level processor stakeholders, who still prioritised primary productivity as highest but ranked water purification and regulation followed by carbon cycling and storage above the nutrient recycling soil function. In general, the carbon cycling and storage and habitat for biodiversity functions represented a lower priority, which may be indicative of potential knowledge gaps. At a policy level, this signals the need for better integration of policies or a potential need to elevate the importance of other soil functions within agricultural policies.

**Fig. 4:**
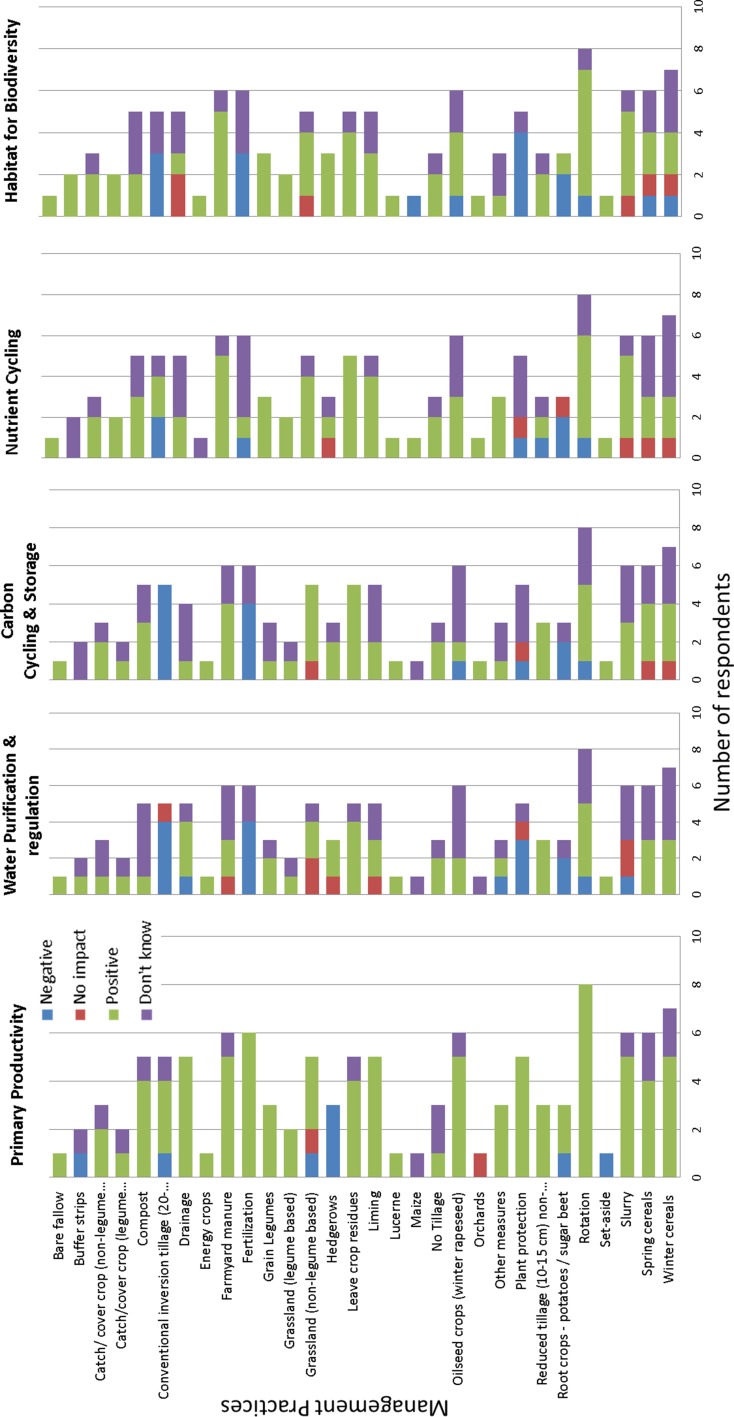
Farmer assessment of management impact on five soil functions delivered through agricultural landscape

Table 3 shows the results of a t-test to compare the entry and exit ranking of soil functions for workshops 5 and 6 combined. This result offers insight into immediate learning effect whilst acknowledging that the learning effect beyond this is not captured here. Results for the water purification and regulation and nutrient cycling functions reflected an increased and decreased significant difference in ranking, respectively.

**Table 3:** Entry and exit ranking of soil functions to quantify immediate learning effect for workshops 5 and 6*

Soil function**	Entry mean (SD)	Exit mean (SD)	df	*P*
Primary productivity	4.31 (± 0.63)	3.64 (± 1.11)	18	0.1
Water purification and regulation	3.02 (± 0.7)	4.0 (± 0.82)	30	0.02
Carbon cycling and storage	2.16 (± 0.81)	2.67 (± 0.82)	20	0.2
Habitat for biodiversity	2.42 (± 0.97)	2.43 (± 0.98)	23	0.98
Nutrient cycling	3.21 (± 0.88)	2.47 (± 0.47)	23	0.04

* A total of 15 marks were available to be assigned over five functions, with a maximum of five marks available for any individual function

** Total respondents *n* = 38

In workshop 7 using a survey instrument, farmers were asked to indicate the impact of management practices on five soil functions. Figure 4 shows that farmer knowledge is strongest for the primary productivity function with knowledge gaps more prevalent across the other four soil functions. With the exception of ‘conventional tillage’, there is limited knowledge indicated on the negative impacts of management on soil functions, and in some instances knowledge may not be accurate, for example, the impact of drainage on the carbon cycling and storage function is rated as positive. Notably, the most ‘don’t know’ responses were for the ‘water purification and regulation’ and ‘carbon cycling and storage’ functions despite national level emphasis on these soil functions in the regulatory landscape in Ireland. This result is consistent with the other workshops (1, 2, 3, 4, 5, 6, 8) whereby a continued need for education and advisory at farm scale to broaden understanding of the capacity and functions of soil beyond primary productivity is identified. This data harvesting has use in identifying knowledge gaps and can support targeting of policies and future education and dissemination efforts.

Notably, these results are reflective of an Irish example; however, when completed for a range of agro-climatic zones the results can support a more targeted approach towards soil function optimisation and sustainable use of the land base. This is based on the assumption that challenges to sustainability vary by location (Schulte et al. 2014) and will be accordingly reflected by stakeholder priorities and captured within the EU LANDMARK project.

#### The Think-Do-Gap

**Fig. 5:**
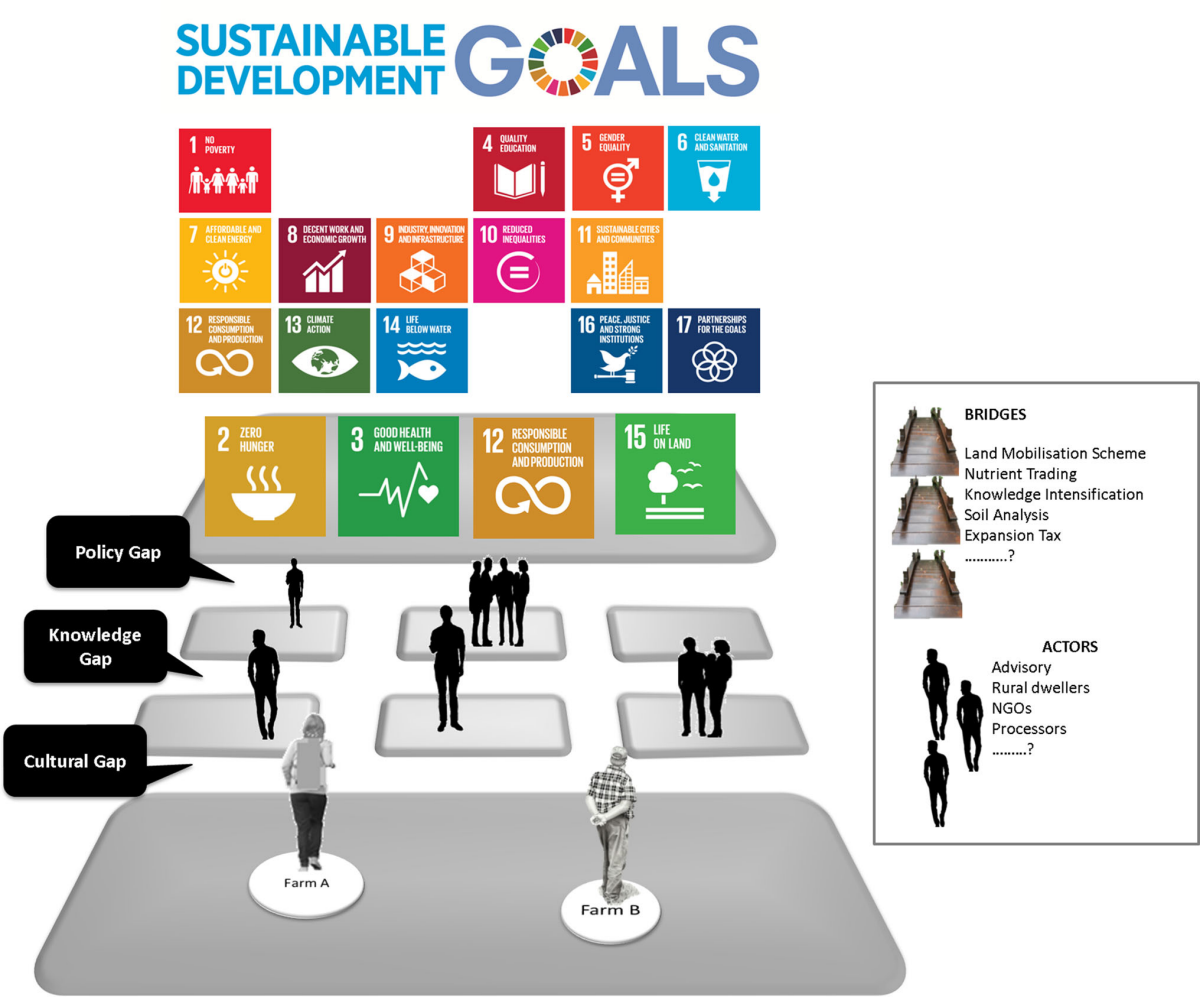
Think-Do-Gap. The sustainable development goals (SDG) represent the global goals to end poverty, fight inequality and tackle climate change (UN 2016) (top from: Communications materials). Four of the SDGs specifically cite soil (2.4, 3.9, 12.4 and 15.3) (UN 2016). The FLM framework is a tool that can be utilised for sustainable agri-environmental development in-line with the SDGs. To transition farmers from their current situation to FLM, governance instruments (bridges) that can steer or incentivise action to bridge gaps must be implemented. However, the governance space includes many diverse actors with a potential role in achieving FLM

This research proposes the catchment challenge method as an important tool to identify solutions and actions necessary to bridge the gap between landscape level implementation of FLM and the scientific research that underpins FLM. This gap between science and implementation is referred to as the Think-Do-Gap (Fig. 5). Using the catchment challenge model, stakeholders were consistently able to design an optimised catchment that could potentially realise the soil function targets set, i.e. ‘Think’ solutions to achieve FLM based on context specific soil, environment and management. Stakeholders were challenged to balance their demands to reach the optimised design. This learning effect was captured not only in the soil functions ranking exercise but in the catchment design which always resulted in a more balanced prioritisation. In this way, the catchment challenges facilitated knowledge production through the identification of more balanced and shared key actions necessary at multiple scales from the local to national scale. The results represent important target areas that require integration into the policy framework to facilitate implementation of FLM that can support more targeted policies based on context-specific social and biophysical conditions. This idea was expanded upon in challenge 2 where participants were asked what instruments would be necessary to support the implementation of the FLM catchment design from challenge 1. For example, the option to ‘lease land’ as proposed in challenge 1 might require a land mobilisation scheme, as identified in challenge 2, to bridge that particular gap (Fig. 5) and so on.

## Further research

Supply and demand for soil functions across the EU will be mapped within the LANDMARK project using large datasets based on biophysical, environment and management data for supply, and policy driver indicators for demand. Beyond this, the workshop data are important to better understand the challenges and opportunities in matching the supply with demand for soil functions from a stakeholder perspective. Thus far, 32 LANDMARK catchment challenge workshops have been facilitated across five partner countries, to gain understanding as to how different soil functions are prioritised associated with location. Although engaging a wide range of stakeholders assumes a greater degree of complexity, this front-end investment in knowledge production can ultimately support more effective long-term change. Often policies represent conflicting goals and agendas which can result in uncertainty for stakeholder application (Carton et al. 2016). Stakeholder engagement can support more coherent policy setting and reduce the risk for unintended consequences to emerge as it includes a much broader consideration of a wide range of value judgements and expertise.

The implementation of FLM requires gaps to be bridged including socio-cultural, bureaucratic and knowledge/education barriers (Fig. 5). Importantly, Fig. 5 represents a starting point for the direction of further research. While all gaps are considered the same in Fig. 5, future research will seek to classify these gaps. For example, policy gaps refer to “institutions”, the so called rules of a game in society that are humanly devised to shape human interaction (North 1990). At a policy level, bridging the gap between science and implementation of FLM might require the introduction of a tax or incentive tool. In contrast, cultural gaps refer to informal rules, but these workshop results indicate that cultural factors are important in shaping agri-environmental governance and are therefore important to understand decision making for the implementation of FLM. Knowledge gaps may refer to technical solutions. While the workshops increase context-specific understanding of the stakeholder challenges and opportunities in relation to soil functions, understanding the societal actors, networks and their interactions is also important. Different actors face different challenges or gaps in the implementation of FLM. A network analysis of the governance space for soil functions in five countries is currently under development within the LANDMARK project. The results from the network analysis are expected to identify existing coalitions or gaps in networks, collaboration opportunities and points of entry that could be targeted to steer stakeholders/decision makers towards FLM.

## Concluding remarks

Although the new SDGs include targets that directly and indirectly relate to soil (UN 2015b), the achievement of these SDGs will remain elusive unless there is inter-disciplinary cooperation between different scientific disciplines along with the continued involvement of stakeholders and policy makers in a trans-disciplinary context (Bouma 2015). With environmental and agricultural policies increasingly framed within a context of ecosystem services, this demand is apparent. As identified in the workshops, the historical approach of utilising single-issue policy measures is likely to be insufficient to achieve multiple objectives from the soil resource. In applied research, a lack of interaction with broader stakeholders groups, such as land managers or policy makers, is likely to result in a breakdown in relation to knowledge production and governance for implementation. Applied research provides an essential foundation towards the validation of policy making; however, it is also important that this research can be scaled up and appropriately translated into policy instruments. Hence, inter-disciplinary scientific input that also considers socio-economics, natural sciences, political science and ethnopedology is likely to result in greater knowledge of systems, and in this case, the gaps and mechanisms to support the delivery of soil functions at a landscape level. In this regard, the trans-disciplinary FLM workshops, through an informal setting, allowed for many value judgements and expertise of a range of stakeholders to be moderated and integrated in a process aimed at informing more effective change. Also, the intrinsic relationship between soil and land means that soil scientists can assume a pivotal role as knowledge brokers in a context of greater inter-disciplinary and trans-disciplinary research (Bouma 2015). With 2015 as the international year of the soil and initiatives such as the 4/1000 for food security and climate change increasing the affinity between society and soils, soil science is well positioned to forward the agenda on sustainable agri-environmental policies.
